# Supercritical CO_2_ Extraction and Microencapsulation of Lycopene-Enriched Oleoresins from Tomato Peels: Evidence on Antiproliferative and Cytocompatibility Activities

**DOI:** 10.3390/antiox10020222

**Published:** 2021-02-02

**Authors:** Liliana Mihalcea, Oana Crăciunescu, Ionica Gheonea (Dima), Ana-Maria Prelipcean, Elena Enachi, Vasilica Barbu, Gabriela Elena Bahrim, Gabriela Râpeanu, Anca Oancea, Nicoleta Stănciuc

**Affiliations:** 1 Faculty of Food Science and Engineering, Dunărea de Jos University of Galati, 800201 Galati, Romania; Liliana.Mihalcea@ugal.ro (L.M.); Ionica.Dima@ugal.ro (I.G.); Elena.Ionita@ugal.ro (E.E.); Vasilica.Barbu@ugal.ro (V.B.); Gabriela.Bahrim@ugal.ro (G.E.B.); Gabriela.Rapeanu@ugal.ro (G.R.); 2 Department of Cellular and Molecular Biology, National Institute of Research & Development for Biological Sciences, 060031 Bucharest, Romania; oana.craciunescu@incdsb.ro (O.C.); anamaria.prelipcean@incdsb.ro (A.-M.P.); anca.oancea@incdsb.ro (A.O.)

**Keywords:** lycopene, supercritical CO_2_ extraction, microencapsulation, antiproliferative activity, cytocompatibility

## Abstract

Tomato peels are used as a valuable material to extract lycopene-rich oleoresins by supercritical CO_2_ extraction. The extraction involves continuous circling of CO_2_ to the extractor after removing the solute in the separators, S40 and S45, where the solvent power of the CO_2_ is reduced by reducing pressure down to 20 MPa in S40 and 5 MPa in S45, respectively, leading to two extracts. Lycopene is found to be the major compound, representing 93% and 76% of the total carotenoids in S40 and S45 extracts, respectively. The two extracts are microencapsulated in whey protein concentrate and acacia gum by complex coacervation and freeze-drying, leading to corresponding P40 and P45 powders, with antioxidant activity of 8.57 ± 0.74 and 9.37 ± 0.48 mMol TEAC/g DW in P40 and P45, respectively. Different structural and morphological patterns are observed, with finer microparticles of 1–2 µm in P45. Both powders show dose and time-dependent antiproliferative activity. The half-maximal inhibitory concentration values are 100 µg/mL for P40 and 750 µg/mL for P45 sample, indicating a higher antiproliferative effect of P40 over P45 in HT-29 cell culture. The powders have an extended range of cytocompatibility, up to 1000 µg/mL, in L929 normal cells, stimulating the cell growth. Lycopene retention is tested, and values of 48% and 29% in P40 and P45 are found after 21 days at 25 °C, with the degradation rate in P45 significantly higher, due to the higher content of the surface lycopene, which favored its degradation.

## 1. Introduction

It is well known that tomato processing is one of the most developed within the vegetable processing industry at an international level, being responsible for important amounts of waste, thus causing a major disposal problem, both in terms of costs and impact on the environment [1]. Colle et al. [2] suggested that millions of tons of tomatoes are processed into products (sauces, juices, soups, and dried tomatoes) or used as ingredients of ready-to-eat meals, resulting in large amounts of by-products, such as peel, pulp, and seeds, representing 10–40% of the total processed tomatoes. A significant part of these by-products is currently being disposed of in the environment or used as animal feed. However, the abundance of lycopene in the peels fraction suggests the possibility of utilizing it as a cheap source for lycopene extraction [3].

The main carotenoid in tomatoes is lycopene, representing approximately 80–90% of the total carotenoids. Due to its open-chain structure with 13 double bonds, with 11 of them conjugated, lycopene has the highest degree of unsaturation among all carotenoids. The presence of the 13 conjugated double bonds gives the characteristic deep red color of ripe tomato fruits and tomato products and the antioxidant activity [4]. Due to its structural particularities, growing demand has been observed for the natural form of lycopene, due to the specific properties to be effective in quenching the destructive free radicals (nitrogen dioxide—NO_2_•, sulfide—RS• and singlet oxygen—O_2_•) and in inhibiting DNA and cellular membrane damage [5]. 

Different benefits associated with the increased dietary consumption of tomatoes and derived products containing lycopene were reported—such as cardiovascular protection, and the decrease of prostate and breast cancer, and coronary heart disease incidence [6], inhibition of the oxidation of low-density lipoproteins, thus, helping to lower the level of blood cholesterol [7], and anti-inflammatory response, by inhibiting the activation of inducible nitric oxide synthase proteins [8]. From a marketing point of view, Scaglia et al. [9] suggested that lycopene is one of ten carotenoids that are currently marketed, with an estimated economic value in 2017 of 1.5 USD billion and an annual growth rate of 2.3%. Therefore, the extraction of the lycopene from tomato waste may bring significant benefits by further introducing the extracts in different foods, cosmeceuticals, and nutraceuticals. However, extractions of the bioactives from food matrices are limited by the requirement of non-toxic solvents that do not remain as residue in the product. Since the lycopene is stored in the tomato peels, it degrades easily during processing [10], supercritical carbon dioxide (SC-CO_2_) extraction has been used as an emerging method to extract tomato oleoresin [11]. SC-CO_2_ extraction is a greener alternative to organic solvent extraction, which operates above the solvent’s critical pressure and temperature, enhancing the solvating power of the solvent. SC-CO_2_ is a solvent frequently used, due to its main advantages as, low critical pressure and temperature, non-toxic, non-inflammable, high purity, low cost, and its availability. 

Precisely due to the high content of unsaturated bonds, responsible for the specific biological properties, isolated lycopene is prone to degradation, being sensitive to oxidants, light, and heat during storage and processing [12], especially in the presence of oxygen [13]. In addition, the application of lycopene in the food industry as an added-value ingredient is drastically limited, due to its characteristics of low solubility in an aqueous environment and low bioaccessibility [14]. Therefore, design strategies are needed to produce lycopene in a stable form, with improved solubility and bioaccessibility. Recently, in order to improve the solubility and bioaccessibility of lycopene, several delivery systems have been developed, such as protein-based nanoparticles [15], emulsions [16], liposomes [17], and microcapsules [18].

Therefore, this study extends the possibility of using tomato peels as a valuable source of lycopene, by SC-CO_2_ extraction and microencapsulation. In the first step, the valorization of fresh tomato peels by supercritical carbon dioxide extraction without modifiers at different extraction parameters, whereas the extracts were characterized in terms of total carotenoids, lycopene content, and antioxidant activity. Further, the extracts with the higher bioactives content were used to produce microcapsules, using whey protein isolates and acacia gum as microencapsulating agents, by complex coacervation and freeze-drying. The resulting powders were tested for microencapsulation efficiency, phytochemicals content and antioxidant activity, structural and morphological appearance, and antiproliferative and cytotoxic properties. In addition, the short-term storage stability of lycopene microcapsules was studied.

## 2. Materials and Methods 

### 2.1. Chemicals

Whey protein isolates (protein content of 95%) was purchased from Fonterra (Auckland, New Zealand). The HPLC analytical-grade hexane, acetone, acetonitrile, ethyl acetate, methanol, and analytical grade [2,2-azinobis-(3-ethylbenzothiazoline-6-sulfonic acid) diammonium salt] (ABTS), 6-Hydroxy-2,5,7,8-tetramethylchroman-2-carboxylic acid (Trolox), acacia gum, lycopene, and β-carotene standards were obtained from Sigma-Aldrich (Steinheim, Germany). Carbon dioxide with a purity of 99.9% vol. was supplied by Messer Romania Gaz SRL (Bucharest, Romania). Dulbecco’s Modified Eagle Medium (DMEM), Minimum Essential Medium (MEM), fetal calf serum (FCS), L-glutamine, a mixture of antibiotics (penicillin, streptomycin, neomycin) (PSN), and neutral red (NR) were purchased from Merck (Germany). HT-29 human colorectal adenocarcinoma cell line was purchased from ATCC, and NCTC clone L929 mouse fibroblast cell line came from ECACC (Sigma-Aldrich, Steinheim, Germany). 

### 2.2. Tomato Fruits

Red ripped tomatoes (*Lycopersicon esculentum* var. Buzău 1600) were purchased at commercial maturity from a local supermarket (Galați, Romania) in August 2019 and stored at 4 °C until processing. The fruits were visually inspected for uniformity of color and selected based on similar characteristics. To obtain peels without any residual pulp, the tomatoes were first washed with distilled water, and the peels were removed manually, washed several times, and then blotted on paper towels to remove any residual pulp. The peels were stored at −20 °C until analyses.

### 2.3. Tomato Peels Supercritical CO_2_ Extraction Process

The extractions with SC-CO_2_ were performed with the pilot plant equipped with an extractor vessel with a total internal volume of 2 L, and two separators S40 and S45, each with a volume of 1.5 L (Natex Prozesstechnologie GesmbH, Ternitz, Austria). SC-CO_2_ flow was monitored with a Coriolis mass flow meter. Several SC-CO_2_ extraction batches have been tested without modifiers. The amount of 0.165 kg of tomato peels, with a moisture content of 79.46 ± 0.41%, was ground and placed in the extractor vessel, and liquid CO_2_ from a cylinder tank with a siphon was passed through a chiller kept at −3 °C and compressed by a high-pressure membrane pump. The solvent was continuously cycled to the extractor after removing the solute in the separators, S40 and S45, where the solvent power of the CO_2_ was reduced by reducing pressure down to 20 MPa in S40 and 5 MPa in S45, respectively. The temperature was set at the same values as in the extractor for the S40 and at 25 °C for the S45, respectively. The extraction parameters were automatically controlled and indicated by ABB software (ABB, Mannheim, Germany). The temperatures of the extractor and both separators were controlled by a surrounding heated jacket, whereas the pressure was controlled by the pressure control valves. Extracting conditions were set as follows: Pressure 40 MPa, temperatures 70 °C, 74 °C and 80 °C and time 155 min according to previous studies [1]. The SC-CO_2_ extracts (named S40 and S45) were collected in vials, then concentrated at 40 °C using a vacuum rotary evaporator (AVC 2–18, Christ, UK), whereas all vials were protected from light and ambient oxygen with aluminum foil to minimize decomposition and oxidation. The obtained extracts were weighed to calculate the gravimetric yield and stored at −20 °C until further analyses. The gravimetric extraction yield was expressed as the ratio between the weight of extracted oleoresin and the weight of matrices loaded in the extractor vessel.

### 2.4. Evaluation of Carotenoids and Lycopene Content in Extracts and Powders

A spectrophotometric method was employed to determine the lycopene and total carotenoid contents in the extracts and powders. Therefore, about 5 mg of dried extracts and 100 mg of each microencapsulated powder were dissolved in 10 mL of a mixture of *n*-hexane:acetone (ratio of 3:1), in a volumetric flask. The absorbance was measured at 470 nm and 503 nm. The amount of lycopene and β-carotene was calculated according to the following equation [13]:

(1) Carotenoid (gL)=A×MW×Df/(Ma×L)

where *A* is absorbance of the hexane phase at 503 and 470 nm; *Mw* is the molecular weight for lycopene, and β-carotene (536.873 and 536.87 g·mol^−1^, respectively), *D_f_* I sample dilution rate, *M_a_* I molar absorptivity in *n*-hexane (3450 L mol^−1^ cm^−1^ for lycopene and 2592 L mol^−1^ cm^−1^ for β-carotene), and *L* I cell diameter of the spectrophotometer (1 cm).

The amount of lycopene and β-carotene were expressed as mg/g DW of extract.

### 2.5. High-Performance Liquid Chromatography (HPLC) Analysis of Lycopene in the Extracts

Based on the spectrophotometric profile, the extract with the higher content in lycopene and β-carotene was selected for HPLC analysis. A Thermo Finnigan Surveyor HPLC system coupled with a DAD UV-visible detector (Finnigan Surveyor LC, Thermo Scientific, USA) was used. The system was controlled by the Xcalibur software (Finnigan Surveyor LC, Thermo Scientific, Waltham, MA, USA), whereas the carotenoids compounds were assessed at 450 nm on a Lichrosorb RP-18 (5 μm) Hibar RT 125–4 column. The mobile phase consisted of two solvents, namely, 90% acetonitrile (A) and 100% ethyl acetate (B). The injection volume was 10 μL, while the flow rate was 1000 mL/min. The elution gradient was: 0–16 min, 15% B; 16–54 min, 15–62% B, 54–56 min, 62% B; 56–60 min, 62–15% B; 60–70 min, 15% B. The quantification of lycopene (LOD 0.05 mg kg^−1^ and LOQ 0.10 mg kg^−1^) and β-carotene (LOD 0.05 mg kg^−1^ and LOQ 0.10 mg kg^−1^) was performed using the calibration curves for each compound. 

### 2.6. Determination of Free Radical Scavenging Activity 

The antioxidant activity of the extracts and powders was measured by using the TEAC (Trolox Equivalent Antioxidant Capacity) assay by evaluating the 2,2-azinobis-3-ethyl-benzothialzoline-6-sulfonic acid (ABTS^+^) radical cation decoloring reaction, as described by Scaglia et al. [9], with slight modifications. Briefly, the working ABTS^∙+^ solution was obtained by mixing the ABTS^∙+^ aqueous solution (7 mM) with potassium persulfate (2.45 mM final concentration), followed by keeping the mixture to react in the dark at room temperature for 16 h. An amount of 5 mg of extracts and 100 mg of powders, respectively, were previously dissolved in 10 mL of a mixture of *n*-hexane:acetone (ratio of 3:1), in a volumetric flask. A volume of 2.85 mL of the ABTS^∙+^ solution was mixed with 0.15 mL of the extract or powders solution and allowed to react for 2 h in a dark room, followed by an absorbance reading at 734 nm. The ABTS^∙+^ antioxidant activity of the samples was expressed as mM TEAC/g DW of extract based on the calibration curve using Trolox.

### 2.7. Microencapsulation of the Extracts by Complex Coacervation and Freeze-Drying

Two extracts were selected for microencapsulation, based on the highest lycopene content, namely, the two fractions corresponding to the two separators (S40 and S45), obtained in the following conditions: 40 MPa, temperature 74 °C, and an extraction time of 155 min. The microencapsulation of the extracts was performed as described by Gheonea (Dima) et al. [18], with modifications. As microencapsulating materials, whey proteins isolate (WPI) and acacia gum were selected and dissolved in distilled water at a ratio of 5:1 (*w*/*w*). The solution was mixed on a magnetic stirrer until complete hydration at 450 rpm and 40 °C. To obtain a coarse emulsion, an amount of 0.5 g of each extract (S40 and S45) was first dissolved in 5 mL of sunflower oil and dripped into the aqueous solution by stirring. The emulsion was then obtained by homogenization using an Ultra Turrax mixer (IKA T18 basic, Staufen, Germany) at 5000× *g* for 10 min. To promote coacervation, the pH of the solutions was adjusted to 3.5 with 1 N HCl solution at approximately 40 °C, under constant mechanical stirring at 600 rpm. The reaction mixtures were cooled in ice bath and stored at 4–6 °C overnight to promote decantation. The coacervates were separated and freeze-dried (CHRIST Alpha 1–4 LD plus, Osterode am Harz, Germany) at −42 °C under a pressure of 10 Pa for 48 h. The resulting powders (coded P40 and P45) were collected and packed in metalized bags and kept in the refrigerator at 4 °C until further analysis. 

### 2.8. Microencapsulation Efficiency

The microencapsulation efficiency was calculated based on Equation (2), involving the measurement of total and free lycopene content. The total and free lycopene content were determined as described by Gheonea (Dima) et al. [18], with slight modifications. The method involved the quantification of total lycopene content (*Lyc_total_*) in the powders and lycopene content on the surface of the microparticles (*Lyc_surface_*). 

*Lyc_total_* were determined by weighing 100 mg of the powders, which were dissolved in 6 mL of 10% NaCl:methanol (ratio of 1:1, *v*/*v*). The solutions were sonicated for 30 min to break the microcapsules, followed by addition of a volume of 30 mL *n*-hexane:acetone mixture (ratio of 1:1, *v*/*v*) and centrifugation at 6000× *g* for 10 min. *Lyc_surface_* involved the use of 50 mg of powders dissolved in 10 mL of 1:1 (*v*/*v*) *n*-hexane:acetone mixture (ratio of 1:1, *v*/*v*). The solutions were agitated for maximum 2 min using a vortex and centrifugated at 1500× *g* for 3 min. In both cases, the optical density of the upper organic fraction was measured at 503 nm. The lycopene content was calculated based on Equation (1). Microencapsulation efficiency was calculated using Equation (2):

(2) $$\mathrm{Microencapsulation}\;\mathrm{efficiency}\;\left(\%\right)=\frac{Lyc_{total}-Lyc_{surface}}{Lyc_{total}}\times 100$$

### 2.9. Structural and Morphological Analysis of the Powders

The structure and the morphology of the obtained powders were determined using a confocal analysis performed on a Zeiss Axio Z1 Observer confocal inverted microscope (Carl Zeiss Microscopy GmbH, Köln, Germany). The LSM 710 microscope uses several laser scanning systems: Diode laser (405 nm), Ar laser (458, 488, and 514 nm), DPSS (561 nm pumped solid-state diodes), and HeNe laser (633 nm). The distribution of bioactives into the complex biopolymer matrix was observed using the 20× apochromatic objective and the 0.6 magnification. The obtained powders were observed both in their native state and fluorescently labeled with Red Congo (40 µM), in a ratio of 3:1. The confocal images of the powders were captured and analyzed with the ZEN 2012 SP1 software (Black Edition, Carl Zeiss Microscopy GmbH, Jena, Germany).

### 2.10. In Vitro Cytotoxicity of the Powders in HT-29 and L929 Cells by Neutral Red Assay

Cell culture tests were performed according to SR EN ISO 10993-5 for medical device cytotoxicity using the direct contact method of cultivation and neutral red (NR) assay for quantitative assessment of cell viability, as previously described [19]. Briefly, HT-29 human colon cancer cells were cultured in DMEM supplemented with 20% (*v*/*v*) FCS, 2 mM L-glutamine and 1% (*v*/*v*) PSN mixture in humidified atmosphere with 5% CO_2_, at 37 °C, until subconfluence. Mouse fibroblasts from NCTC clone L929 cell line were cultured in MEM supplemented with 10% (*v*/*v*) FCS, 2 mM L-glutamine and 1% (*v*/*v*) PSN mixture in humidified atmosphere with 5% CO_2_, at 37 °C, until subconfluence. For the experiment, HT-29 cells and L929 cells were cultured in 24-well microplates, at a density of 4 × 10^4^ cells/mL, in specific culture media, and the plates were incubated in a humidified atmosphere with 5% CO_2_, at 37 °C for 24 h, in order to allow cell adhesion to the plastic substrate. Then, the culture medium was replaced by a serially diluted stock solution of the microencapsulated powders, to give final concentrations between 1–1000 µg/mL, and the incubation continued in standard conditions, for 24 and 48 h, respectively. At the end of each incubation period, the conditioned medium was replaced with 50 μg/mL NR solution, and the plate was incubated at 37 °C, for 3 h. After cell washing, the incorporated dye was released in 1% (*v*/*v*) acetic acid solution in 50% (*v*/*v*) ethanol by gentle shaking, for 15 min. The amount of up taken dye was directly proportional to the number of viable cells. The optical density (OD) was recorded at 540 nm in a Spectrostar nano microplate reader (BMG Labtech, Germany). The results of cell viability were expressed as a percentage from untreated cells (control) using the following equation:

Cell viability (%) = OD_sample_/OD_control_ × 100 (3)

### 2.11. Light Microscopy

Cell morphology of the control and powders-treated cultures was observed by light microscopy for the qualitative evaluation of the powders cytocompatibility. After 48 h of cultivation in the presence of the microencapsulated powders, the cells were washed, fixed in methanol, and Giemsa stained. Micrographs were acquired at an Axio Observer D1 microscope equipped with a digital camera (Carl Zeiss, Germany).

### 2.12. Stability Study 

For storage stability study, the powders were kept in light-resistant glass bottles at 25 ± 1 °C for 21 days and observed for any change and percent residual content of lycopene.

### 2.13. Statistical Analysis of Data 

All the experiments were performed in triplicates, with duplicate samples. The results were expressed in terms of average values. Statistical analysis of the data was performed by univariate analysis of variance (ANOVA) with a significance level of 95% (*p* < 0.05) using the Tukey’s test. 

## 3. Results

### 3.1. The Phytochemical Content and Antioxidant Activity of the Tomato Peels Extract

In the first extraction batch, freshly grinded peels with value (%) of the dry matter of 20.54 ± 0.41% were extracted using SC-CO_2_ at the following parameters: Pressure 40 MPa, different temperature of 70 °C, 74 °C, and 80 °C, and the CO_2_ masic flow of 0.32 kg/min. The phytochemicals content of the extracts obtained in different separators is given in Table 1. 

The different phytochemical profiles, in terms of lycopene and β-carotene showed in Table 1, may be explained by temperature effect on the extraction rate of carotenoids. Therefore, for the extract separated at 20 MPa in the first separator S40, it can be observed that the increase of the extraction temperature, from the 70 °C to 80 °C led to the increase of the lycopene content from 6.06 ± 0.06 mg/g DW to 8.11 ± 0.65 mg/g DW, respectively. This can be explained by the effect of the temperature on the improvement of the mass transfer and the solvent diffusion through the ground vegetable matrix. Likewise, the β-carotene concentrations were positively correlated with the increase of extraction temperature from 70 °C to 74 °C in both fractions. At the extraction temperatures of 70 °C, the gravimetric yield was in the range of 3.39% for first separation S40 and 6.18% at the second separator S45, respectively. At the higher extraction temperature up to 80°C, the gravimetric yield was at 3.81% in the first separator S40 and 5.99% in the second one, S45, respectively.

Our results could be explained according to the separation conditions after the extraction process. Therefore, at the same extraction pressure of 40 MPa, the fractionating of the oleoresins was performed using two consecutive separators with controlled temperature and pressure conditions. Our results indicate that at 5 MPa in the second separator, S45, recovers the higher lycopene and β-carotene content. From Table 1 it can be seen that, overall, a higher content in lycopene and β-carotene was obtained when extracting at a temperature of 74 °C, leading to an extract with a lycopene content of 39.11 ± 0.59 mg/g DW and β-carotene of 68.24 ± 0.71 mg/g DW in S45 separator. Machmudah et al. [20] reported a lycopene concentration of 459.20 mg/kg at 40 MPa at 90 °C and a CO_2_ flow rate of 4 mL/min for 180 min. The antioxidant activity was higher for the extract separated at 20 MPa with 77.61 ± 0.99 mM TEAC/g DW in S40 separator. Due to the highest concentration values of bioactive, it has been found that the extraction conditions of temperature 74°C, pressure 40 MPa, 155 min and SC-CO_2_ flow rate 0.32 kg/min allowed to obtain a gravimetric extraction yield of 2.12% in the first separator (pressure 20 MPa and temperature 74 °C) and 8.06% for the second separator (pressure 5 MPa and the temperature 25 °C), respectively. 

**Figure 1:**
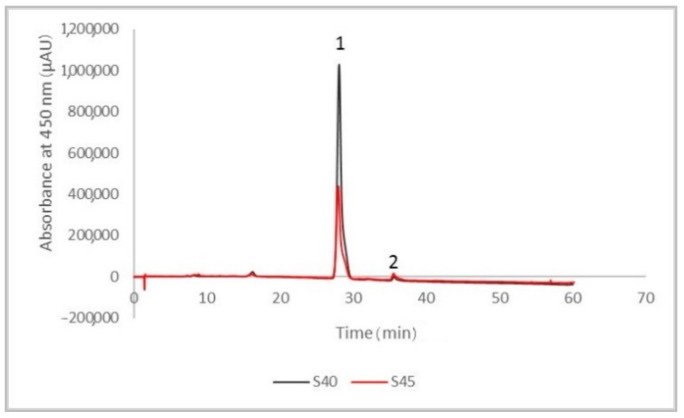
The chromatographic profile of the tomato peels supercritical CO_2_ extracts at 450 nm (S40—the extract fraction separated at 20 MPa in the first separator, S45—the extract fraction separated at 5 MPa in the second separator), the major identified compounds being lycopene (peak 1) and β-carotene (peak 2).

Figure 1 shows the chromatographic profile of the two selected extracts, obtained at the best SC-CO_2_ extraction condition of pressure 40 MPa, temperature 74 °C, and extraction time of 155 min.

The compounds were separated and identified based on their retention time and the calibration curves and by comparison with the data reported in the literature. The major compounds identified were lycopene (peak 1) and β-carotene (peak 2). For the S40 extract, the content of lycopene was 92.23% (corresponding to a concentration of 0.875 mg/mL) from the total carotenoids, whereas the content of β-carotene was 1.51% (corresponding to a concentration of 0.015 mg/mL). The results obtained for the S45 extract were significantly lower compared to S40 extract, as such, the content of lycopene was 76.36% (corresponding to a concentration of 0.345 mg/mL), whereas the content of β-carotene was 4.78% (corresponding to a concentration lower than 0.010 mg/mL) of the total carotenoids content. Our results were similar to those reported by Rizk, El-Kady, and El-Bialy [21], who stated that lycopene was the major carotenoid compound extracted from tomatoes, with a content of 86.13% of the total carotenoids, whereas β-carotene displayed content of about 2.11%. Moreover, Ooe et al. [22] determined the Japanese blue tomato carotenoids content and identified two major compounds, lycopene (with a content of 1.13 mg/g extract) and β-carotene (0.10 mg/g), respectively.

### 3.2. Microencapsulation Efficiency of the Selected Extracts 

High encapsulation efficiency is needed to reduce oxidative degradation of lycopene on the particle surface and to increase powders’ stability [23]. In our study, similar values for microencapsulation efficiency of lycopene in both powders, with values of 41.06 ± 0.33% for P40 and 40.42 ± 0.59% for P45, respectively. Jia et al. [14] encapsulated lycopene in whey protein isolate-xylo-oligosaccharide conjugates prepared by Maillard reaction, reporting significantly higher encapsulation efficiency and encapsulation of 94% and 86%, respectively, at specific conditions to prepare the conjugates of protein-oligosaccharide ratio of 1:2, heating time 3 h and pH 9.0. Jain et al. [24] established maximum entrapment efficiencies (63.1 ± 3.6% for lycopene) at 1:2 of Gum tragacanth:casein and 0.75 wt% of total biopolymers concentration, by complex coacervation. These authors suggested that on increasing the quantity of lycopene loaded oil, while keeping the total polymeric strength constant, % entrapment efficiency increased up to a certain level. Microcapsules obtained with 2.5 mL of oil containing lycopene led to a bioactive entrapment of 66.6 ± 24.5%.

### 3.3. Powders Phytochemical Profile and Antioxidant Activity

Lycopene content in the particles produced with WPI and acacia gum were of 10.83 ± 0.09 mg/g DW in P40 and of 10.49 ± 0.23 mg/g DW in P45 (Table 1). Souza et al. [13] reported a lycopene content varying from 333.7 to 494.4 μg/g, using as encapsulating agent’s maltodextrin, WPI, and the modified starch Capsul^®^. The antioxidant activity of the powders showed similar values of 8.57 ± 0.74 mMol TEAC/g DW in P40 and 9.37 ± 0.48 mMol TEAC/g DW in P45 (Table 1). 

### 3.4. Structural and Morphological Properties of the Powders

**Figure 2:**
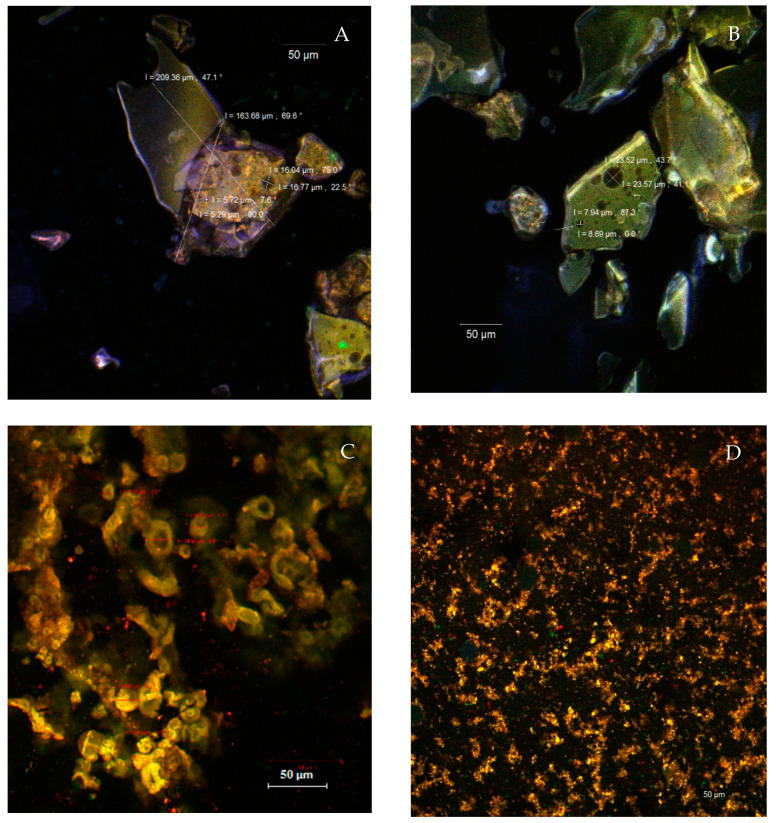
The confocal laser scanning microscopy (CLSM) images (20× apochromatic objective, zoom 1) of the unstained P40 (**A**) and P45 (**B**) native powders and the fluorophore dyed P40 (**C**) and P45 powders (**D**).

The prepared microscope slides from the native powders (Figure 2A,B) indicate the microencapsulation of the phytochemical compounds from the tomato peels in the form of large, polygonal scales, with dimensions between 163.68–209.36 µm inside of which spherical vesicles (spherosomes) with diameters of 5.29–16.77 µm (in P40) or 7.94–23.5 µm (in P45) can be observed. As the major compound in the extract, the lycopene, with an autofluorescence in the range of 500–580 nm, was captured in the microencapsulating matrix formed by WPI (in green) and acacia gum (in blue). The results were similar to those obtained in the native state by Neagu et al. [25], who microencapsulated oleoresin supercritical extracts from sea buckthorn into WPI and casein.

By fluorescent labeling, various significant differences between the two powders were highlighted (Figure 2C,D). Thus, the P40 powder formed several spherosomes, with sizes ranging between 15.87 μm and 38.54 μm, showing a coalescent tendency. In the P45, the ultrastructural aspect was different, highlighting the presence of fine microparticles with sizes of 1–2 µm, with an emission predominantly in the yellow domain and aggregated into coacervates. The fluorophore bounded to WPI, incorporating lycopene, which caused the fluorescent emission migration to the green-yellow range (560–580 nm), more obvious in the P45 powder. The larger size of the microcapsules in the P40 was correlated with better stability over time, better retention of the valuable phytochemicals, and better antioxidant activity.

### 3.5. Antiproliferative Activity of the Microencapsulated Powders

**Figure 3:**
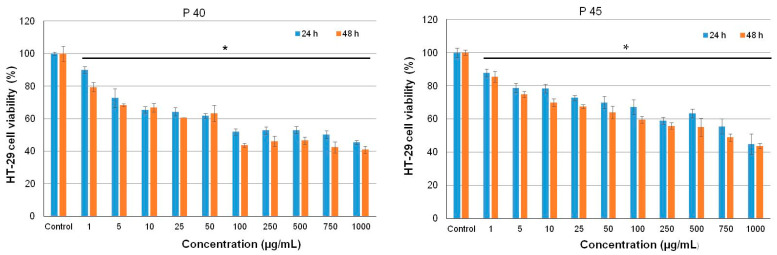
Cell viability of HT-29 cells cultivated in the presence of different concentrations of P40 and P45 microencapsulated samples, for 24 h and 48 h, assessed by NR assay. The results were expressed as mean ± SD (*n* = 3). * *p* < 0.05, compared to untreated control.

*In vitro* cytotoxicity of the microencapsulated powders was assessed in HT-29 and L929 cells, after 24 and 48 h of cultivation by NR assay. The results showed a dose-dependent decrease of HT-29 cell viability for both tested powders (Figure 3).

P40 significantly inhibited (*p* < 0.05) cell proliferation of HT-29 cells in the range of concentrations 5–1000 µg/mL with 30 to 60% at 24 h of cultivation. At 48 h of cultivation, the same descending pattern was observed, but slightly higher values were recorded for each concentration value and maximum inhibition of 55% at 1000 µg/mL. Similar, P45 induced 25–55% inhibition of HT-29 cell proliferation, in the range of concentrations between 25–1000 µg/mL, at both 24 and 48 h of incubation, showing both dose- and time-dependent antiproliferative activity.

**Figure 4:**
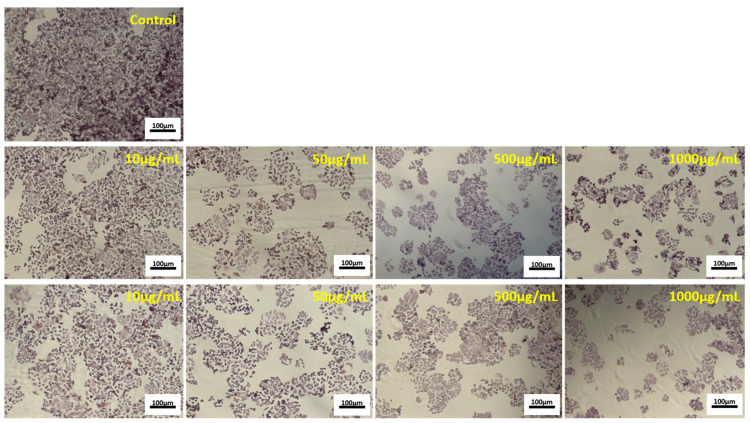
Representative light micrographs of HT-29 cells cultivated in the presence of different concentrations of P40 (second line) and P45 (third line) microencapsulated materials, for 48 h. The untreated culture served as control (first line). (Giemsa staining). Bar scale = 100 µm.

The half-maximal inhibitory concentration (IC50) values were 100 µg/mL for P40 and 750 µg/mL for P45, indicating a higher antiproliferative effect of P40 over P45 microencapsulated powders in HT-29 cell culture. Cell morphology observations followed NR quantitative data (Figure 4). 

The micrographs showed that increasing concentrations of P40 and P45 powders decreased the cell density in HT-29 treated cultures, compared to the control culture, in a dose-dependent manner. It was observed that treated cells maintained their normal aggregative phenotype, but their viability and proliferation were severely affected. The same concentrations of P40 and P45 microencapsulated powders were tested for possible toxic compounds release in normal L929 cells by NR assay.

**Figure 5:**
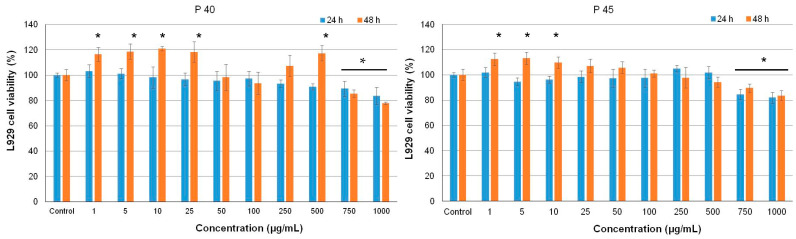
Cell viability of L929 cells cultivated in the presence of different concentrations of S40 and S45 microencapsulated materials, for 24 h and 48 h, determined by NR assay. The results were expressed as mean ± SD (*n* = 3). * *p* < 0.05, compared to untreated control.

The results showed no cytotoxicity of both samples, in the range of concentrations 1–1000 μg/mL (Figure 5). 

Thus, the values of cell viability varied between 77.9–116.4% for P40 and 83.6–112.4% for P45, at 48 h of cultivation. Significantly lower values (*p* < 0.05) of cell viability were recorded only for 750 and 1000 μg/mL concentrations, compared to untreated control cells (100% cell viability), but they were within the non-cytotoxic domain (80–100% cell viability), except for 1000 μg/mL P40 at 48 h of cultivation (77.9%). In addition, concentrations in the range of 1–500 µg/mL P40 and 1–10 µg/mL P45 significantly stimulated (*p* < 0.05) cell proliferation of normal cells, compared to the control culture, after 48 h of cultivation.

**Figure 6:**
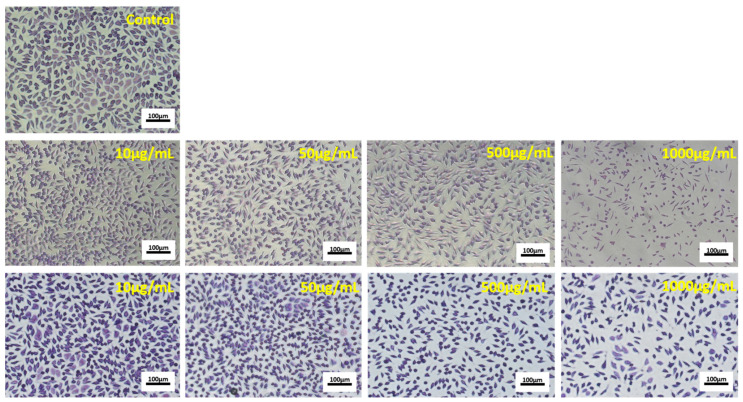
Representative light micrographs of L929 cells cultivated in the presence of different concentrations of P40 (second line) and P45 (third line) microencapsulated materials, for 48 h. The untreated culture served as control (first line). (Giemsa staining). Bar scale = 100 µm.

Light micrographs showed that cell morphology and density observations of L929 treated with different concentrations of microencapsulated powders were in accordance with NR quantitative data (Figure 6). 

The images showed that treated fibroblasts maintained their characteristic fusiform phenotype and were homogeneously distributed on the culture plate, similar to the control culture, for all tested concentrations. At low concentrations (1–100 µg/mL), the cell density was higher than that of control culture, while at high concentrations (750–1000 μg/mL), the cell density moderately decreased in treated cultures. All these data demonstrated that the novel encapsulated powders exhibited antiproliferative activity in tumor cells, while extending the range of cytocompatibility in normal cells. They indicated that P40 and P45 microencapsulated powders had exerted an antiproliferative effect at minimal concentrations of 5 µg/mL and 25 µg/mL, respectively, in HT-29 cells, after 24 h of cultivation. At the same time, it is worth noticing that these powders presented an extended range of cytocompatibility, up to 1000 µg/mL, in L929 normal cells and even stimulated the cell growth.

Previous studies on standard lycopene have tested its effect on cell proliferation, at physiological concentrations or higher than 0.5 µg/mL, in several non-tumoral and tumoral cell lines. A wide range of low lycopene concentrations ranging between 0.00005–0.5 µg/mL showed minimal inhibition of cell proliferation in IMR-90 normal lung fibroblasts culture, and cell viability decreased by only 10%, after 48 h of cultivation [26]. In vitro cultivation of 0.5–3 µg/mL lycopene in several human cancer cell lines showed an inhibitory effect of HT-29 cells with 20% and T84 cells with 30%, after 48 h of cultivation, due to its internalization and induction of changes in the cell cycle, but no effect on other cell types (A549, DU145, HepG2, Hela, Hep-2) [27]. A lycopene-rich extract of red guava, encapsulated in 200 nm lipid nanoparticles or not, showed good cytocompatibility in NIH-3 T3 normal mice fibroblasts and decreased the viability of MCF-7 human adenocarcinoma cells down to 30%, at 100 µg/mL, thus indicating antiproliferative activity, while protecting healthy cells [28]. No studies on the antiproliferative activity of lycopene encapsulated in a protein-polysaccharide complex were found.

Recent studies have shown that lycopene administration together with doxorubicin was able to attenuate the drug’s cardiotoxicity and to improve its antitumor activity [29]. Lycopene is a carotenoid known as a potent antioxidant agent, while doxorubicin is a chemotherapy drug belonging to anthracyclines class. It was supposed that the role of lycopene was mainly as a preventive factor against the formation of cancer cells, and its mechanism of action was different from that of doxorubicin in tumor cells. Several in vitro studies showed that lycopene inhibited intercellular gap junction, the progression of cell cycle and cell invasion, and important signaling pathways within tumor cell cultures, acting also as an antiproliferative agent [30].

### 3.6. Retention Rate of Lycopene at Storage Test

The retention rate of the core material is an important indicator to measure the storage stability of microencapsulated lycopene. In this study, the retention rate of the lycopene was studied in dark and 25 °C, for three weeks. The retention rate of lycopene in the microcapsules was approximately 48% and 29% in P40 and P45, respectively, after storage for 21 days at 25 °C. Jia et al. [14] reported similar values for the retention rate of lycopene in the conjugate microcapsules of 46% and 40% after storage for 36 days at 25 °C and 40 °C, respectively. The degradation of lycopene in powders during storage was fitted to a first-order kinetic model. The degradation rate of lycopene in P45 is significantly higher when compared with P40, the *k* values for the first-order kinetic model being of 85.44 ± 1.23 × 10^−3^ days^−1^ for lycopene degradation in P40 and significantly higher, of 144.16 ± 2.56 × 10^−13^ days^−1^ in P45. The different pattern of lycopene degradation in P45 is due to the higher content of the surface, non-encapsulated lycopene, when compared with P40, which enhanced the degradation of lycopene by oxidation and isomerization. The half time of total lycopene content was 8.11 ± 0.94 days in P40 and 4.80 ± 0.34 days in P45. Surface carotenoids were reduced to one-half in P40 in 13 days, whereas in P45, more than 78% indicated the sensitivity of lycopene toward oxidation, isomerization, and degradation despite moderate storage temperature and the exclusion of light.

## 4. Conclusions

In this study, two complementary approaches were employed for recovery and uses of bioactives from tomato peels to develop added-value ingredients with multiple uses. Therefore, supercritical fluid extraction parameters were optimized to obtain extracts with superior characteristics, followed by microencapsulation and testing of resulting powders. The SC-CO_2_ extraction of tomato peels by-product at a temperature of 74 °C, pressure of 40 MPa, and extraction time of 155 min yielded high amounts of lycopene-enriched oleoresin. The two extracts corresponding to the equipment separators were microencapsulated in whey protein isolates and acacia gum by complex coacervation and freeze-drying, resulting in two powders with similar microencapsulation efficiency, phytochemicals content, and antioxidant activity. However, although using the same microencapsulation technique, the powders showed different morphological, stability, and biological properties. The ultrastructural aspect highlighted the presence of different sizes of spherosomes, with a coalescent tendency in the powder containing extract separated at 20 MPa, whereas finer microparticles up to 2 µm, aggregated into coacervates were observed in the powder containing the extract obtained in the second separator at 5 MPa. A significant effect of cell proliferation in a normal cell at concentrations up to 500 µg/mL for the powder with the extract separated at 20 MPa, and up to 10 µg/mL for the powder with the extract separated at 5 MPa was observed, whereas an antiproliferative effect at minimal concentrations of 5 µg/mL and 25 µg/mL, respectively, in HT-29 cells was highlighted. Both powders presented an extended range of cytocompatibility, up to 1000 µg/mL, in L929 normal cells. The degradation of lycopene in powders during storage was fitted to a first-order kinetic model, highlighting a higher degradation rate of lycopene in the powder with the extract separated at 5 MPa, namely, due to the higher content of the surface, non-encapsulated lycopene, thus enhancing its degradation by oxidation and isomerization.

Our results are promising in opening new opportunities to identify sustainable solutions for the development of value-added ingredients with multiple uses, such as food, pharmaceutics, cosmeceuticals, and nutraceuticals.

## Figures and Tables

**Table 1:** The phytochemicals content of the extracts, obtained in different separators (S40 and S45) and the corresponding microencapsulated powders.

Matrix	Phytochemicals					
	Lycopene (mg/g DW)		β-Carotene (mg/g DW)		Antioxidant Activity (mM TEAC/g DW)	
	S40	S45	S40	S45	S40	S45
SCE extract (70 °C)	6.06 ± 0.06 ^b^	9.48 ± 0.41 ^c^	10.88 ± 0.33 ^b^	18.93 ± 0.73 ^c^	48.52 ± 3.63 ^b^	24.35 ± 0.71 ^c^
SCE extract (74 °C)	5.28 ± 0.07 ^b^	39.11 ± 0.59 ^a^	12.57 ± 0.11 ^a^	68.24 ± 0.71 ^a^	77.61 ± 0.99 ^a^	62.74 ± 1.74 ^a^
SCE extract (80 °C)	8.11 ± 0.65 ^a^	30.59 ± 0.63 ^b^	11.85 ± 0.59 ^ab^	47.08 ± 1.05 ^b^	39.99 ± 1.02 ^c^	38.34 ± 2.13 ^b^
Microencapsulated powder (P40)	10.83 ± 0.09 ^A^		22.49 ± 1.00 ^A^		8.57 ± 0.74 ^A^	
Microencapsulated powder (P45)	10.49 ± 0.23 ^A^		19.93 ± 0.42 ^B^		9.37 ± 0.48 ^A^	

SCE—supercritical CO_2_ fluids extraction. The results were expressed as mean ± SD (*n* = 3). Means that do not share the same letter (a, b, c for the extracts and A and B for the powders) are significantly different (*p* < 0.05).

## Data Availability

HT-29 human colorectal adenocarcinoma cell line was purchased from American Type Culture Collection (ATCC), and NCTC clone L929 mouse fibroblast cell line came from European Collection of Authenticated Cell Culture (ECACC) (Sigma-Aldrich, Steineim, Germany).
